# Novel dental resin infiltrant containing smart monomer dodecylmethylaminoethyl methacrylate

**DOI:** 10.3389/fcimb.2022.1063143

**Published:** 2022-11-15

**Authors:** Xiaoyu Huang, Jingou Liang, Wen Zhou, Tao Ma, Michael D. Weir, Gary D. Hack, Guadalupe Garcia Fay, Thomas W. Oates, Lei Cheng, Hockin H. K. Xu

**Affiliations:** ^1^ State Key Laboratory of Oral Diseases, National Clinical Research Center for Oral Diseases, West China School of Stomatology, Sichuan University, Chengdu, China; ^2^ Department of Operative Dentistry and Endodontics, West China Hospital of Stomatology, Sichuan University, Chengdu, China; ^3^ Department of Advanced Oral Sciences and Therapeutics, University of Maryland Dental School, Baltimore, MD, United States; ^4^ Stomatology Hospital, School of Stomatology, Zhejiang University School of Medicine, Zhejiang Provincial Clinical Research Center for Oral Diseases, Key Laboratory of Oral Biomedical Research of Zhejiang Province, Cancer Center of Zhejiang University, Hangzhou, China; ^5^ Department of Pediatric Dentistry, West China School of Stomatology, Sichuan University, Chengdu, China; ^6^ Fujian Key Laboratory of Oral Diseases & Fujian Provincial Engineering Research Center of Oral Biomaterial & Stomatological Key lab of Fujian College and University, School and Hospital of Stomatology, Fujian Medical University, Fuzhou, China; ^7^ Department of Oncology and Diagnostic Sciences, University of Maryland School of Dentistry, Baltimore, MD, United States; ^8^ Center for Stem Cell Biology & Regenerative Medicine, University of Maryland School of Medicine, Baltimore, MD, United States; ^9^ Marlene and Stewart Greenebaum Cancer Center, University of Maryland School of Medicine, Baltimore, MD, United States

**Keywords:** quaternary ammonium, dodecylmethylaminoethyl methacrylate, dental caries, resin infiltrant, white spot lesion

## Abstract

**Objectives:**

White spot lesions (WSLs) are prevalent and often lead to aesthetic problems and progressive caries. The objectives of this study were to: (1) develop a novel resin infiltrant containing smart monomer dodecylmethylaminoethyl methacrylate (DMAEM) to inhibit WSLs, and (2) investigate the effects of DMAEM incorporation on cytotoxicity, mechanical properties, biofilm-inhibition and protection of enamel hardness for the first time.

**Methods:**

DMAEM was synthesized using 1-bromododecane, 2-methylamino ethanol and methylmethacrylate. DMAEM with mass fractions of 0%, 1.25%, 2.5% and 5% were incorporated into a resin infiltant containing BisGMA and TEGDMA. Cytotoxicity, mechanical properties and antibacterial effects were tested. After resin infiltration, bovine enamel was demineralized with saliva biofilm acids, and enamel hardness was measured.

**Result:**

DMAEM infiltration did not increase the cytotoxicity or compromise the physical properties when DMAEM mass fraction was below 5% (*p* > 0.05). Biofilm metabolic activity was reduced by 90%, and biofilm lactic acid production was reduced by 92%, *via* DMAEM (*p* < 0.05). Mutans streptococci biofilm CFU was reduced by 3 logs (*p* < 0.05). When demineralized in acid and then under biofilms, the infiltrant + 5% DMAEM group produced an enamel hardness (mean ± sd; n = 6) of 2.90 ± 0.06 GPa, much higher than 0.85 ± 0.12 GPa of the infiltrant + 0% DMAEM group (*p* < 0.05).

**Significance:**

A novel resin infiltrant with excellent mechanical properties, biocompability, strong antibacterial activity and anti-demineralization effect was developed using DMAEM for the first time. The DMAEM resin infiltrant is promising for inhibiting WSLs, arresting early caries, and protecting enamel hardness.

## 1 Introduction

Dental caries is one of the most prevalent oral diseases globally (Peres et al., 2019). White spot lesions (WSLs) are early-stage caries, which have been reported to affect between 50% and 96% of patients receiving fixed orthodontic treatments (Sardana et al., 2019; Sonesson et al., 2020). Treatments for patients suffering from WSLs include fluoride, phosphopeptide compounds, and resin infiltration (Wegehaupt and Attin, 2010; Leila et al., 2017). Resin infiltant can penetrate into the pores of enamel and seal the passage of acid. It is minimally invasive and has been an emerging therapeutic modality in the treatment of WSLs. However, resin infiltant can only seal off about 30-60% of the enamel pore volume (Kielbassa et al., 2009; Yim et al., 2014). Furthermore, after the attack of acid produced by cariogenic microorganisms, the hardness of enamel decreased significantly (Dai et al., 2022).

In the oral cavity, dental caries starts with the breakdown of the dynamic balance of oral microecology (Pitts et al., 2017). During the development of caries, oral microbial diversity is decreased, and the acidogenic bacteria can multiply and produce acids, causing the pH to decrease and leading to hard tissue demineralization (Selwitz et al., 2007). Antibacterial agents such as silver nanoparticles (AgNP) (Kielbassa et al., 2020; Li et al., 2020), quaternary ammonium methacrylate (Yu et al., 2020), and ionic liquid-loaded microcapsules (Cuppini et al., 2021) were incorporating into dental materials to inhibit the growth of plaque. However, oral commensal microbiome colonizes on dental tissue plays an essential role in oral microecology (Rosier et al., 2018). The oral probiotics were also inhibited by the agents mentioned above, that would break the balance of oral microecology (Pitts et al., 2017; Liang et al., 2020). Therefore, an intelligent resin infiltrant that could show an antibacterial effect only during dysbiosis, rather than killing all the bacteria, is highly preferred to inhibit WSLs.

Recently, microecosystem-regulating effects of intelligent pH-sensitive resin materials have received more attention (Fenton et al., 2018). Dodecylmethylaminoethyl methacrylate (DMAEM) is a novel tertiary amine (TA) smart material that responds to pH change. Its central nitrogen atom is connected to 3 alkyl or aromatic groups. When the local pH is low, such as during biofilm acid attacks, the nitrogen atoms of TA is protonated to form quaternary ammonium salts (QAMs), which are antibacterial. The QAMs-modified dental materials demonstrated excellent antibacterial effect (Han et al., 2017; Wang et al., 2019; Zhou et al., 2019). In previous studies, the MIC and MBC of DMAEM against *Streptococcus mutans* UA159 (*S. mutans*), *Streptococcus gordonii* DL1 (*S. gordonii*), and *Streptococcus sanguinis* SK1 (*S. sanguinis*) under different pH were tested by serial microdilution assays (Liang et al., 2020). When the pH was 5, the MIC of DMAEM against *S. mutans*, *S. sanguinis* and *S. gordonii* were 0.18 μg/mL, 0.37 μg/mL, and 5.95 μg/mL, respectively. The MBC against *S. mutans*, *S. sanguinis* and *S. gordonii* were 1.4 μg/mL, 2.9 μg/mL, and 11.9 μg/mL, respectively. These values indicate a strong antibacterial activity at pH 5.

However, when the pH was 7.4, the MIC and MBC of DMAEM became much higher, at more than 13.5 mg/mL, which meant much lower antibacterial activity (Liang et al., 2020). Therefore, DMAEM was strongly antibacterial only when pH was low and when tooth-protection was most needed. These results indicate that DMAEM was smart and had a pH-sensitive capability (Liang et al., 2020). In addition, the smart DMAEM-modified resin adhesives could successfully combat secondary caries *in vivo* and *in vitro* (Liang et al., 2020). Furthermore, smart DMAEM sealants showed the potential to reduce microleakage, thus preventing dental caries (Li et al., 2021).

To date, there has been no report on pH-sensitive modification of resin infiltrant to inhibit WSLs. Considering the unique pH-responsive feature of DMAEM, which was suitable for the unique acidic environment of WSLs, we designed an intelligent pH-sensitive resin infiltrant containing DMAEM for the first time. The objectives of the present study were to: (1) develop a novel intelligent resin infiltrant containing smart monomer dodecylmethylaminoethyl methacrylate (DMAEM) to inhibit WSLs, and (2) evaluate the cytotoxicity, mechanical properties, biofilm-inhibition and protection of enamel hardness of the novel resin infiltrant. The following hypotheses were tested: (1) Novel resin infiltrant containing DMAEM could be successfully synthesized; (2) DMAEM resin infiltrant could inhibit biofilm growth and acid production; and (3) DMAEM resin infiltration could protect enamel and retain its hardness after biofilm acid attacks.

## 2 Methods and materials

### 2.1 Synthesis of DMAEM and preparation of specimen

According to the work described previously, DMAEM was synthesized and verified (Liang et al., 2020; Li et al., 2021). In brief, 100 mmol of 1-bromododecane was added to 500 mmol of 2-methylamino ethanol in 80 mL isopropanol at room temperature. After stirring for 8-10 h under reflux at 85°C, the mixture was cooled to room temperature and slowly poured into 150 mL of diethyl ether. Then the mixture was washed with deionized water and brine, dried over anhydrous Na_2_SO_4_ and vacuumized. Then 31.8 mL methylmethacrylate, catalyst CAA (107 mg, 0.4 mol%) and polymerization inhibitor methoxyphenol (100 mg, 2 mol%) were added at room temperature. After stirring at 100-110°C for 12 h, CAA (150 g, 0.6 mol%) was supplementary, and then stirred for another 12 h, cooled to room temperature and evaporated.

According to the study published before, the experimental resin infiltrant contains bisphenol-Adiglycidyl methacrylate (BisGMA, Esstech; Essington, PA, USA), triethylene glycol dimethacrylate (TEGDMA, Esstech; Essington, PA, USA), and a light cure initiator system based on camphorquinone (CQ) and ethyl 4-N,N-dimethylaminobenzoate (4E) (Hashemian et al., 2021; Prodan et al., 2022). DMAEM was mixed with the experimental resin infiltrant in the dark, at a DMAEM mass fraction of 1.25%, 2.5%, 5% and 7.5%, respectively (**Table 1**). And a magnetic stirrer stirred the mixture constantly for 24 h in a yellow room. The infiltrant + 0% DMAEM group served as the control group.

**Table 1:** Component of experimental resin infiltrant (%).

Materials	BisGMA	TEGDMA	CQ	4E	DMAEM
Infiltrant + 0% DMAEM (Control)	24	75	0.5	0.5	0
Infiltrant + 1.25% DMAEM	23.7	74.06	0.49	0.49	1.25
Infiltrant + 2.5% DMAEM	23.4	73.13	0.49	0.49	2.5
Infiltrant + 5.0% DMAEM	22.8	71.25	0.48	0.48	5
Infiltrant + 7.5% DMAEM	22.2	69.375	0.4625	0.4625	7.5

### 2.2 Cell viability test

A 96-well plate (Costar; Corning Inc., NY, USA) was used as a mold for the samples (Yu et al., 2020). 5 μL of resin infiltrant were added to each well, and then light-cured for 40 s (Triad 2000; Dentsply, York, PA, USA). To remove the uncured monomers, all the samples were immersed in deionized water for 24 h. The samples were sterilized by ethylene oxide in an ethylene oxide sterilizer (Anprolene AN 74i; Andersen, Haw River, NC, USA). Samples of the same group (n = 6) were immersed in 200 μL of cell medium and soaked at 37 °C for 24 h to obtain the extracts, which were collected and diluted to 10 mL, and the extract was diluted to 36 times, 64 times, 128 times with fresh medium (Zhang et al., 2013; Zhang et al., 2013). The NOKSI immortalized normal oral keratinocyte cell line (provided by Dr. Tao Ma, University of Maryland) were cultured and maintained in keratinocyte serum-free medium with growth factor supplement (Gibco; Thermo, Carlsbad, USA) and 1% antibiotic/antimycotic (AA) (Millipore Sigma; Merck KGaA, Darmstadt, Germany) at a density of 40,000 cells per mL. Cells were cultured at 37°C and 5% CO_2_ (Wisniewski et al., 2021). The negative control group was inoculated into the medium without extract. All groups were replaced with fresh medium after 24 h. After 48 h of incubation, 10 μL of CCK-8 solution (Cell Counting Kit-8; Dojindo, Kumamoto, Japan) was added and incubated at a constant temperature incubator for one hour. Optical density (OD) was measured at a wavelength of 450 nm using a microplate reader (SpectraMax M5; Molecular Devices, Sunnyvale, CA, USA). Six replicates were tested for each group.

### 2.3 Mechanical properties test

Rectangular molds (2 × 2 × 25 mm) were used for mechanical testing (Cheng et al., 2012). After composite bars were immersed in distilled water at 37 °C for 1 d, a computer-controlled Universal Testing Machine (5500R; MTS, Cary, NC, USA) was used to test the mechanical properties. In brief, the specimens were fractured in three-point flexure with a 10-mm span at a crosshead-speed of 1 mm/min. Flexural strength (S) was calculated as: S = 3PmaxL/(2bh2), where Pmax is the fracture load, L is span, b is specimen width, and h means specimen thickness. And, elastic modulus (E) was calculated as: E = (P/d) (L3/[4bh3]), where load P divided by displacement d is the slope of the load-displacement curve in the linear elastic region. Six specimens were tested for each material (n = 6).

### 2.4 Bacterial inoculation

Saliva derived biofilm was used in the study. Ten healthy individuals without active caries with natural dentition served as donors. Donors did not take any antibiotics in the three months previous to donation and fasted for two hours. The saliva was diluted two-fold with sterile 50% glycerol. Then the saliva was stored at -80 °C (Li et al., 2017).

Each well of 96-well plate with resin infiltrant was added with 300μL Mcbain medium (Yu et al., 2020). The saliva-glycerol stock was seeded (1:50 final dilution) into plates and incubated under aerobic environment at 5% CO_2_ with 37 °C. The medium was refreshed every 12 h. After 24 h, phosphate-buffered saline (PBS) was used to rinse the biofilms to remove loose bacteria.

### 2.5 Anti-bacterial test

#### 2.5.1 Biofilm accumulation

A crystal violet assay was performed to analyze biomass accumulation (Huang et al., 2019). Briefly, each group included six duplicate samples. After the biofilm was rinsed with PBS, 200 μL 95% methyl alcohol was added to each well and incubated for 15 min to fix the biofilm. After the biofilm was rinsed with PBS, submerging in 300 μL 0.1% crystal violet solution for 30 min. Then, washing with PBS. Finally, 300 μL 95% ethanol solution was added to each well and the plate was shaken at 80 rpm for 45 min at room temperature. Subsequently, 100 µL ethanol of the solution from each well was transferred to a 96-well plate, and a microplate reader was used to measure the absorbance of the solution at a wavelength of 595 nm.

#### 2.5.2 MTT test

The biomass accumulation reflects the whole biofilm which contains live cells and dead cells. It is important to evaluate the metabolic activity that was produced by live cells only. In the present study, the MTT (3-(4,5-Dimethyl-thiazol-2-yl)-2,5-diphenyltetrazolium bromide) assay was used to measure metabolic activity (Zhou et al., 2020). Each group involved six duplicate samples. After proliferation for 24 h, the biofilms were rinsed with PBS and 200 μL MTT dye was added to each well (0.5 mg/mL MTT in PBS). Then, the biofilm plates were cultured for 1 h at 37 °C. After removing the MTT solution, 300 μL dimethyl sulfoxide (DMSO) was added and shaken at 80 rpm for 20 min in the dark to dissolve the formazan crystals. Finally, 100 µL of DMSO was placed into a 96-well plate, and the absorbance was read at a wavelength of 540 nm using a microplate reader.

#### 2.5.3 Lactic acid test

First, biofilms were rinsed by cysteine peptone water (CPW) to remove loose bacteria (Cheng et al., 2012). And 300 μL of buffered peptone water (BPW) supplemented with 0.2% sucrose was added, and incubated at 5% CO_2_ with 37°C for 3 h to produce acid. After 3 h, the BPW solution was used for lactate analysis. An enzymatic (lactate dehydrogenase) method was used to evaluate the lactate concentrations. Microplate reader was used to measure the absorbance at 340 nm for the collected BPW solutions. Standard curves were prepared using a lactic acid standard (Supelco Analytical; Bellefonte, PA, USA).

#### 2.5.4 Live/dead staining

BacLight Live/Dead Bacterial Viability Kit (Molecular Probes; Eugene, OR, USA) was used in the study (Huang et al., 2021). Live bacteria cells were stained with SYTO 9 to produce green fluorescence, and cells with compromised membranes were stained red by propidium iodide. The disks were examined using inverted epifluorescence microscope (TE2000-S; Nikon, Melville, NY, USA), and percent of live bacteria (%) was calculated by ImageJ software (ImageJ_v1.8.0; National Institutes of Health, USA). Percent of live bacteria (%) = live bacteria/(live bacteria + dead bacteria). Six replicates were tested for each group.

#### 2.5.5 CFU

After 24 h incubation, biofilms were harvested for colony-forming unit (CFU) analysis (Cheng et al., 2016). Three types of agar plates were prepared. First, tryptic soy blood agar culture plates were used to determine the total number of microorganisms. Second, mitis salivarius agar (MSA) culture plates, containing 15% sucrose, were used to determine the total number of *streptococci*. Third, MSA agar culture plates plus 0.2 units of bacitracin per mL were used to determine the number of *mutant streptococci* (Zhang et al., 2012).

### 2.6 Enamel hardness test

For anti-demineralization test, bovine teeth were used as previously described (Yu et al., 2020). In brief, bovine permanent incisors free of lesions and cracks were selected. Crowns were cut into sections measuring 4 × 4 × 2 mm by using a diamond-coated band saw with continuous water cooling (Isomet; Buehler, Lake Bluff, IL, USA). These surfaces were then ground flat with water-cooled carborundum discs made of waterproof silicon carbide paper (Extec; Enfield, CT, USA) with various grits (1000, 1200, 2400, 3000, and 4000) (Cheng et al., 2008; Iizuka et al., 2014; He et al., 2015). To remove the residual abrasives, all of the polished samples were individually sonicated in distilled water for five minutes.

**Figure 1:**
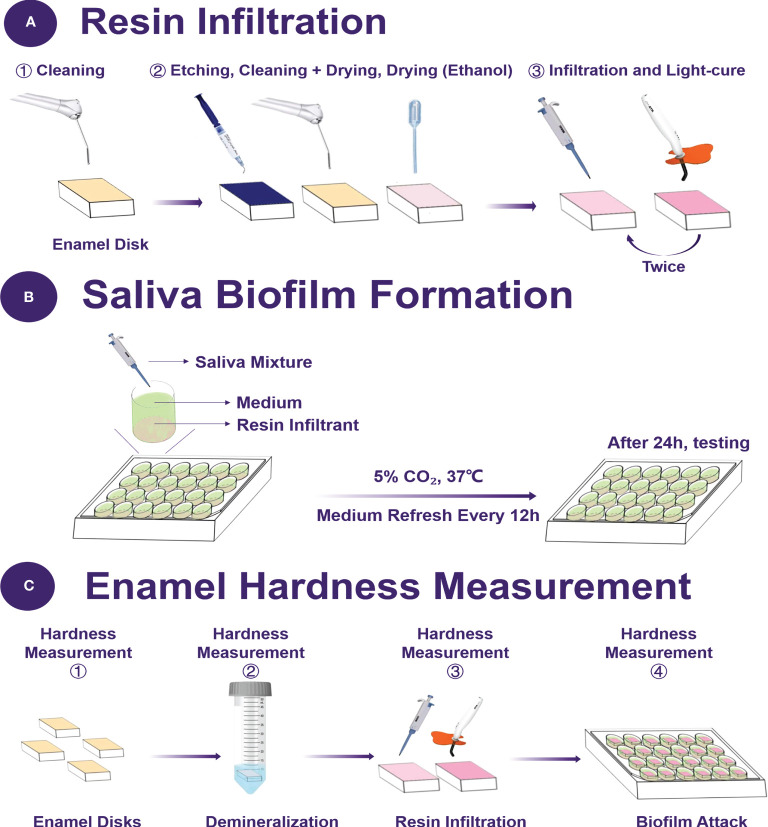
Schematic of the experimental design. **(A)** The protocol of resin infiltration, **(B)** the saliva biofilm formation on the resin disks, **(C)** the time point of enamel hardness measurement.

Initial enamel caries was produced in enamel blocks, as described earlier (Rocha Gomes Torres et al., 2011). The demineralization solution contained 75mM acetic acid (pH 4), 8.7 mM Ca(Cl)_2_, 8.7 mM KH_2_PO_4_, and 0.05 ppm NaF (acetic acid, Ca(Cl)_2_, KH_2_PO_4_, NaF; Biofroxx, Hessen, Germany) (Gao et al., 2020). The blocks were immersed in the demineralization solution (8 mL per block) at 37°C for 16 h with stirring. After dried, 37% phosphoric acid was coated on the demineralization site of the samples for two minutes. Then, rinsed with water for 30 s, and the samples were dried with an air syringe without oil or water. In the demineralization area, 1 μL 99% ethanol was used to dehydrate the area thoroughly. After 30 s, the areas were dried with a water-free and oil-free air syringe. Resin-infiltrant (1 μL) was applied to the demineralized zone for three minutes. After removing the excess material, the area was light-cured for 40 s. Afterward, infiltrant was applied again. After one minute, the excess material was removed again and light-cured for 60 s (**Figure 1**). All samples were sterilized in an ethylene oxide sterilizer.

Enamel hardness was measured before the demineralized treatment, after demineralization in acid solution, after resin infiltration and after demineralization under biofilms. A hardness tester (Shimadzu HMV-2000, Kyoto, Japan) was employed using a Vickers diamond indenter with a load of 25 g for 5 s dwell time (Liang et al., 2019; Gao et al., 2020). Six indentations were made in each enamel specimens, and each group had six specimens.

### 2.7 Statistical analysis

Statistical analyses were performed with SPSS, version 22.0 (SPSS; Chicago, IL, USA). One-way analysis of variance (ANOVA) was performed to detect the significant effects of the variables. Tukey’s multiple comparison test was used to compare the means of each group at a p-value of 0.05.

## 3 Results

**Figure 2:**
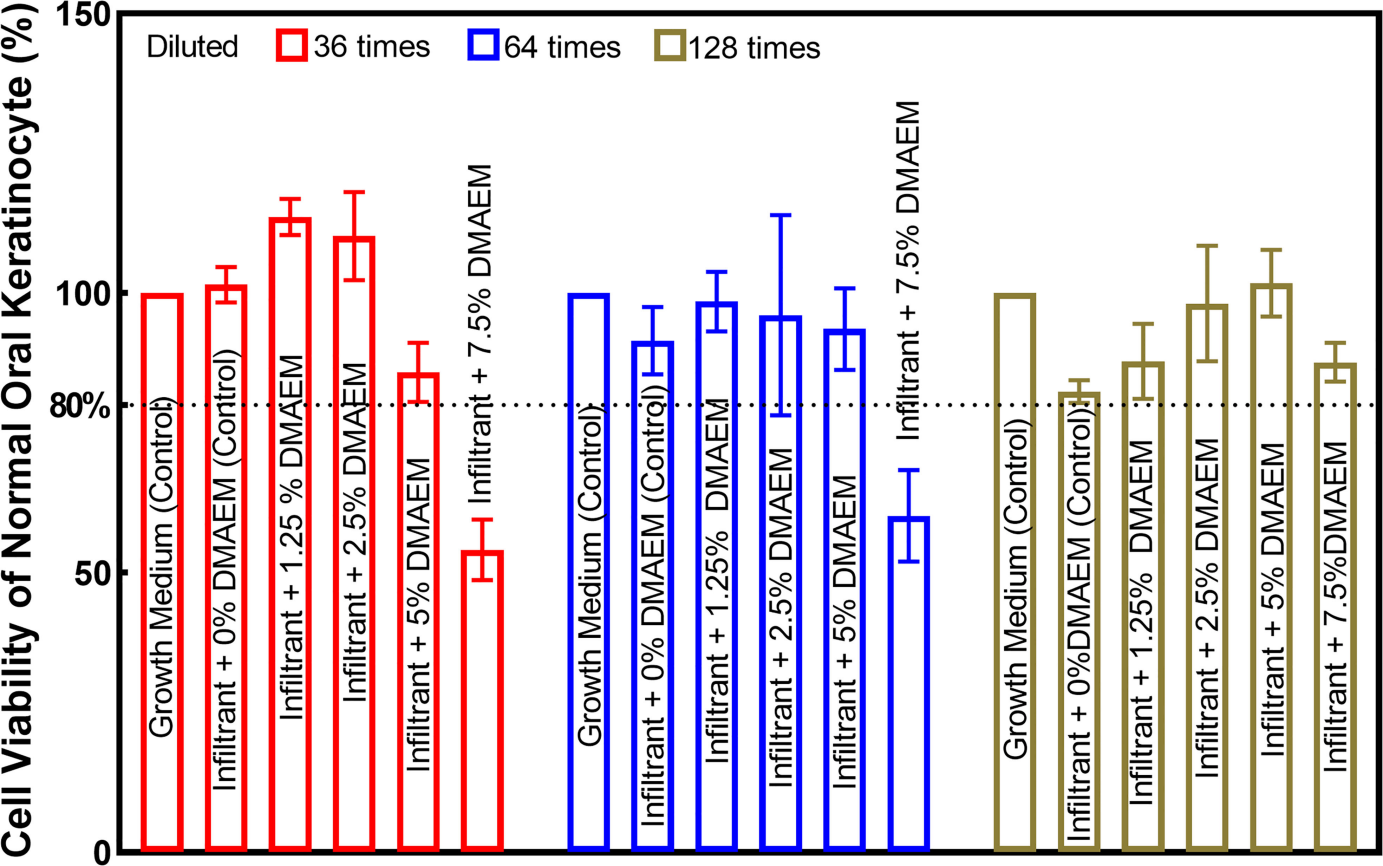
Cell viability of normal oral keratinocyte was used to test the biocompability of DMAEM resin infiltrant. The viability was acceptable when that was not less than 80% of the control group according to ISO 10993-5-2016 standard.

Cell viability results of resin infiltrant are plotted in **Figure 2**. Results showed that all the groups except for infiltrant + 7.5% DMAEM group (*p* < 0.05) had acceptable cell viability (*p* > 0.05).

**Figure 3:**
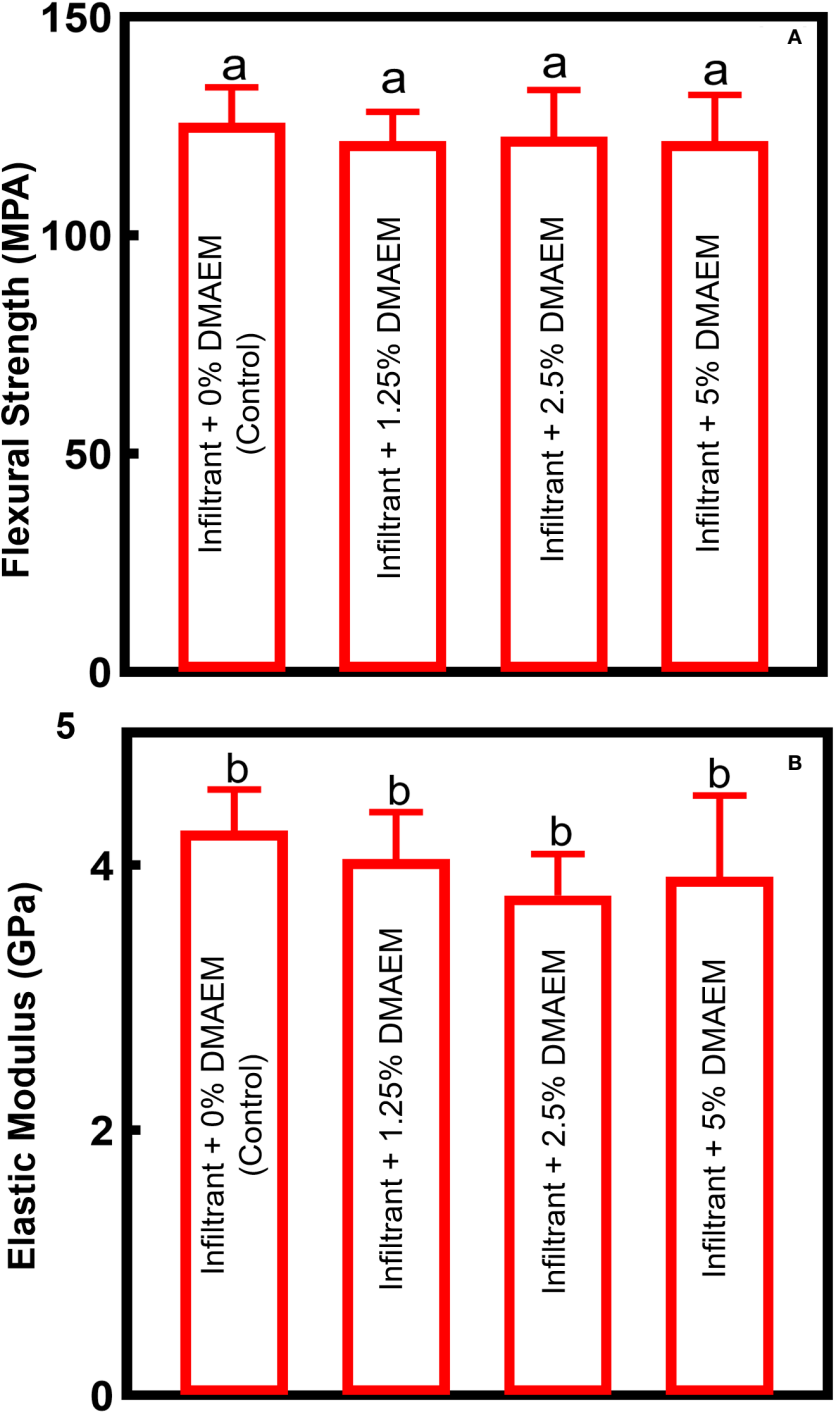
Mechanical properties of DMAEM resin infiltran. **(A)** The flexural strength, **(B)** the elastic modulus (mean ± SD; n = 6). The different letters indicate the significant difference between the bars (a, b, c), there were no significant difference among the four groups (p > 0.05).

Mechanical properties of resin infiltrant are shown in **Figure 3**. All DMAEM resin infiltant had flexural strength and elastic modulus comparable to the infiltrant + 0% DMAEM group (control), which are around 130 MPa and 4 GPa respectively (*p* > 0.05). The DMAEM modification did not affect the mechanical properties of resin infiltrant.

**Figure 4:**
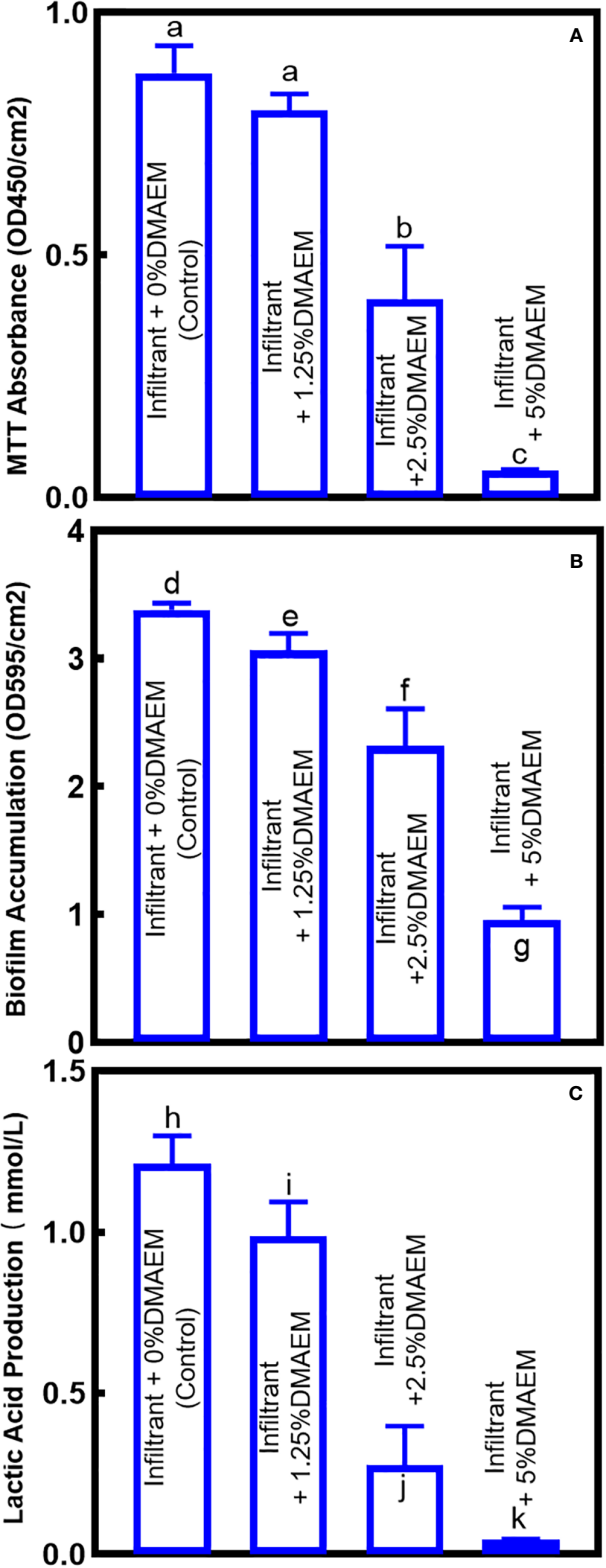
Antibacterial effects of composites on saliva-derived biofilm. **(A)** The biofilm metabolism evaluation, **(B)** biofilm accumulation, **(C)** production of lactic acid of the biofilm sites (mean ± SD; n = 6). The different letters indicate the significant difference between the bars (a, b, c).

**Figure 4** showed the quantification of biofilm viability. MTT assay showed that the metabolism of biofilm decreased with the increase of DMAEM concentration (*p* < 0.05), while the infiltrant + 1.25% DMAEM group didn’t show significant difference (*p* > 0.05). Crystal violet assay and lactic acid production assay plotted similar results, with the increase of DMAEM concentration, the biofilm accumulation and the lactic acid production decreased (*p* < 0.05).

**Figure 5:**
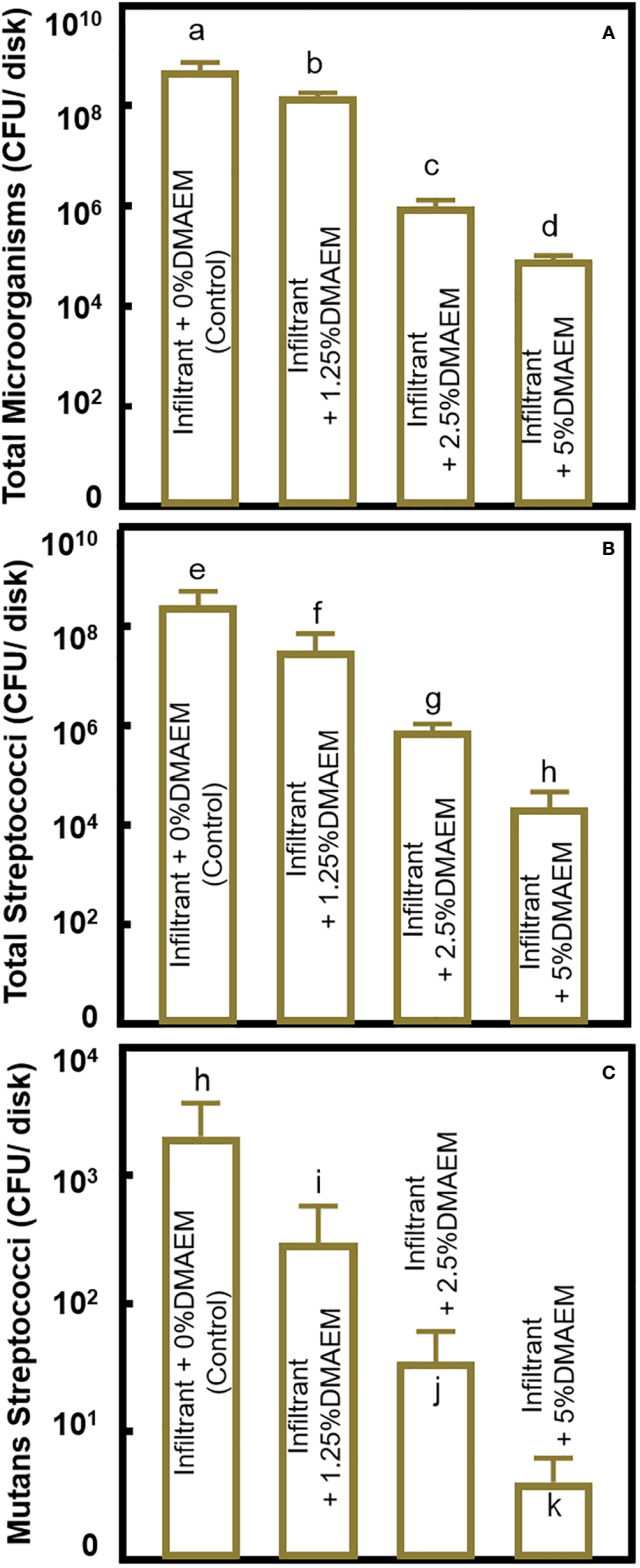
CFUs of the biofilm. **(A)** CFU of total microorganisms, **(B)** CFU of total *streptococci*, **(C)** CFU of *mutans streptococci* (mean ± SD; n = 6). The different letters indicate the significant difference between the bars (a, b, c).

CFU is plotted in **Figure 5**. For total microorganisms, compared to the infiltrant + 0% DMAEM group (control), the infiltrant + 1.25% DMAEM group reduced around 1 log, the infiltrant + 2.5% DMAEM group reduced more than 2 logs, and the infiltrant + 5% DMAEM group reduced more than 3 logs (*p* < 0.05). CFU has reduced the total *streptococci* counts by around 1 log from the infiltrant + 0% DMAEM group (control) to the infiltrant + 1.25% DMAEM group, by more than 2 logs to the infiltrant + 2.5% DMAEM group, and by more than 4 logs to the infiltrant + 5% DMAEM group (*p* < 0.05). For *mutans streptococci* counts, the infiltrant + 1.25% DMAEM group reduced around 1 log, the infiltrant + 2.5% DMAEM group reduced around 2 logs, and the infiltrant + 5% DMAEM group reduced more than 3 logs compared to the control group (*p* < 0.05).

**Figure 6:**
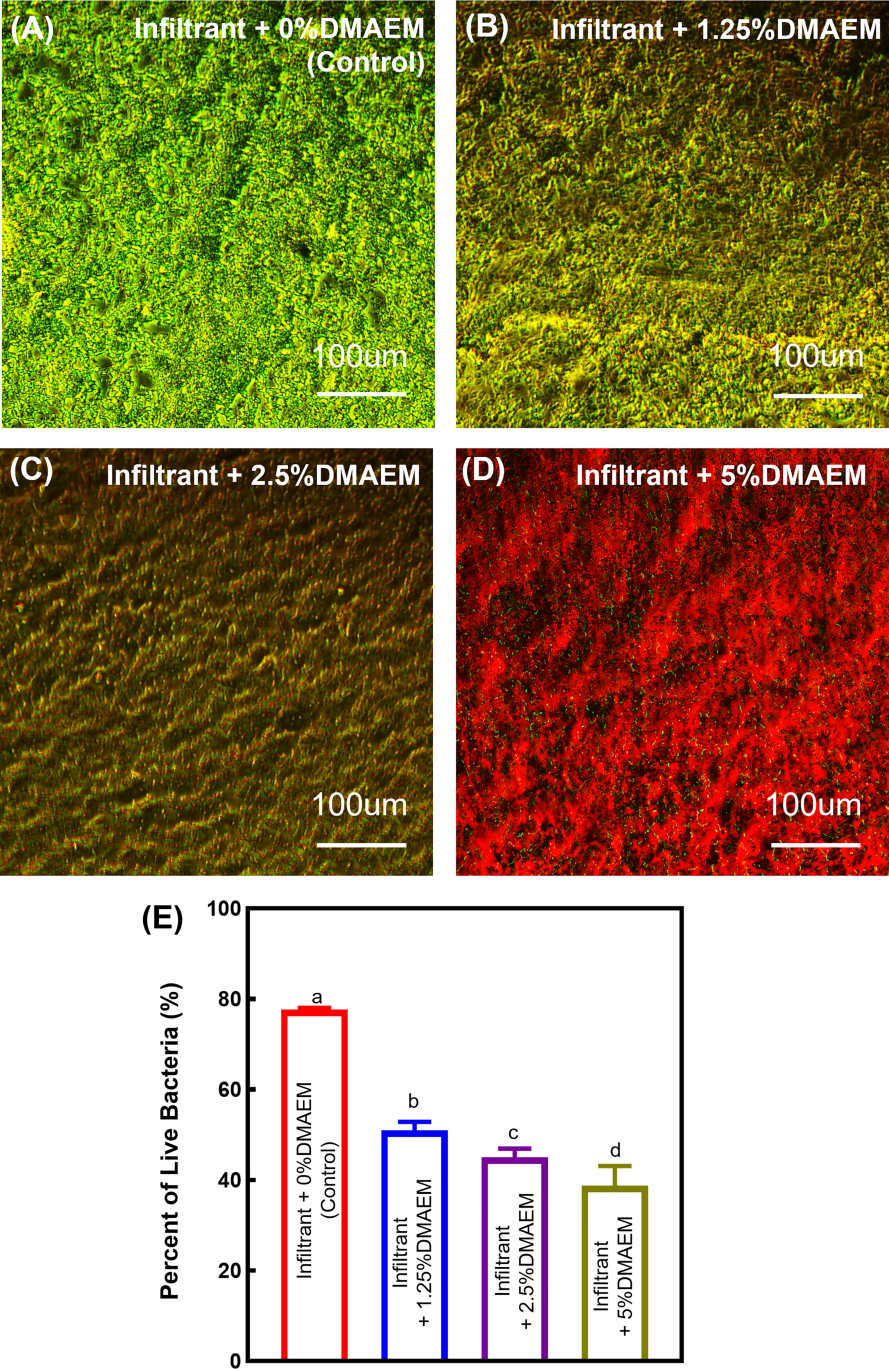
Representative images of live/dead stained biofilms grown for 24 h on composites. **(A)** Infiltrant + 0% DMAEM group, **(B)** infiltrant + 1.25% DMAEM group, **(C)** infiltrant + 2.5% DMAEM group, **(D)** infiltrant + 5% DMAEM group, **(E)** percent of live bacteria(%). Live bacteria were stained green and dead bacteria were stained red. Infiltrant + 0% DMAEM group had primarily live bacteria, while infiltrant + 5% DMAEM group produced mostly red staining.

Live/dead staining images (**Figure 6**) showed that the infiltrant + DMAEM groups had much more red staining, indicating a strong anti-bacterial effect, while the infiltrant + 0% DMAEM group (control) had more green staining and little red staining, which represented little anti-biofilm activity. **Figure 6E** showed live/dead ratio, the infiltrant + DMAEM groups had lower percent of live bacteria (*p* < 0.05), and the percent of live bacteria decreased with increasing DMAEM concentration (*p* < 0.05).

**Figure 7:**
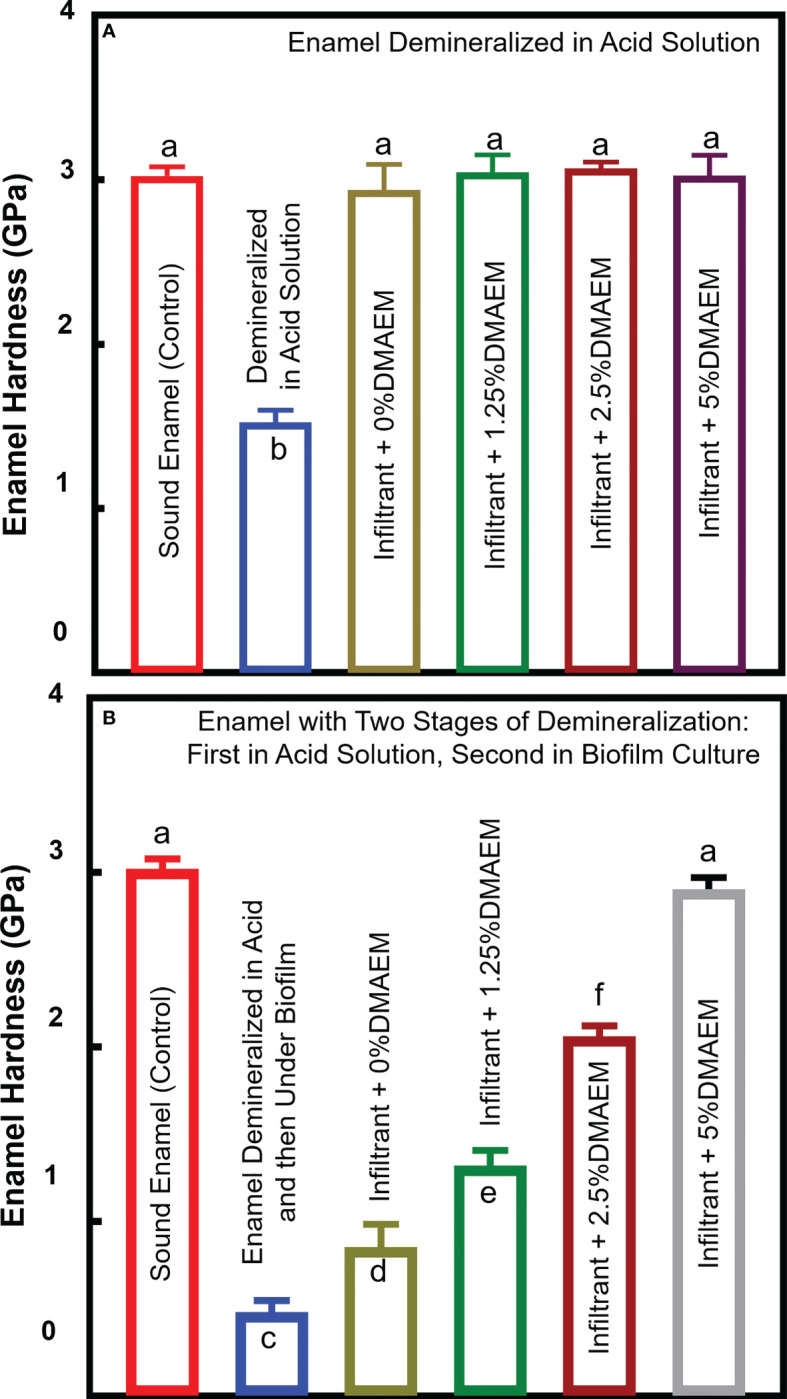
Enamel hardness. **(A)** Enamel samples were demineralized in acid solution, after then, infiltrant was applied as Figure 1. **(B)** Enamel samples were first demineralized like **(A)** and after infiltration, all the samples were second demineralized under biofilm (mean ± SD; n = 6). The different letters indicate the significant difference between the bars (a, b, c). The resin infiltration showed similar hardness value of sound enamel (p > 0.05), and after biofilm attack, only the hardness of the infiltrant + 5% DMAEM group did not decrease (p > 0.05).

Enamel hardness test is plotted in **Figure 7**. Results showed that the hardness of enamel decreased after being demineralized in acid solution (*p* < 0.05), while the resin infiltration recovered the hardness (*p* > 0.05) (**Figure 7A**). After 48 h biofilm demineralization, the hardness of all the groups except for the infiltrant + 5% DMAEM group decreased (*p* < 0.05). After two stages of demineralization: first in acid solution, second in biofilm culture, the enamel showed the lowest hardness in all the group (*p* < 0.05). While the hardness value increased as the increasing of the DMAEM concentration (*p* < 0.05) (**Figure 7B**).

## 4 Discussion

In the present study, a novel pH-sensitive DMAEM infiltrant with antibacterial and anti-demineralizing properties was developed. The hypotheses were proven that the new resin infiltrant had the acceptable mechanical properties without jeopardizing the biocompability, which had normal oral keratinocyte viability similar to that of the infiltrant + 0% DMAEM group (control). Additionally, the antibacterial effect enhanced the anti-demineralizing properties under the attack of biofilm for 7 days.

New materials used for dental treatment are required to be non-cytotoxic and biocompatible. In the present study, cytotoxicity test was carried out according to ISO 10993-5-2016 standard (Li et al., 2015). According to the ISO standard, *in vitro* cytotoxicity tests, the MTT value is not less than 80% prove to be slightly cytotoxic (Li et al., 2015). NOKSI were used in the present study because they are clinically relevant and in close proximity to dental restorations. Our findings suggest that the new resin infiltrant did not induce any significant toxicity when the mass fraction of DMAEM was below 5%, which was similar to the previous study (Liang et al., 2020). Therefore, the concentration of subsequent experiments was selected. These findings indicate that DMAEM resin infiltrant is suitable for clinical applications. Future *in vivo* experiments are needed to investigate the biocompatibility of the infiltrant containing DMAEM.

Mechanical properties of resin infiltrant may influence the hardness of the enamel, as well as the longevity of treatment (Paris et al., 2013). To evaluate whether DMAEM would jeopardize the mechanical properties, we made the resin infiltrant into a cuboid with the size of 2 × 2 × 25 mm. The result demonstrated that the modification did not affect the mechanical properties of resin infiltrant (*p* > 0.05). In detail, flexural strength of the resin infiltrant was around 120-130 MPa, while the elastic modulus was about 4 GPa, which were similar to other results (Prodan et al., 2022). The DMAEM could covalently grafted to resin infiltrant by reaction of acrylate groups with methacrylate groups, yielding the non-release antibacterial dental material. The covalent binding explained the similar mechanical properties of infiltant + DMAEM groups to the control group. The mechanism was similar to that of the DMADDM-modified and DMAHDM-modified dental resins, which had been reported before (Han et al., 2017; Cheng et al., 2017).

WSLs are early-stage of dental caries, which are common side effects of orthodontic treatments (Paula et al., 2017). WSLs are caused by cariogenic biofilm, leading to demineralization of the enamel (Zou et al., 2018). In detail, during the dental caries, the oral microbial diversity is decreased, cariogenic microorganisms proliferate, whereas pH value decreases in a certain period of time (Zou et al., 2018). The balance of demineralization and re-mineralization is shifted to mineral loss, finally the caries is formed (Zou et al., 2018). Therefore, biofilm inhibition is the first step to solve WSLs (Abdullah and John, 2016; Horst, 2018).

The resin infiltration concept was first developed in the 1970’s (Cheng et al., 2017), low viscosity resin materials had been used to restore decalcified enamel at that time (Paula et al., 2017; Zou et al., 2018), such as sealant and adhesive. At the end of the 2000’s, with the development of dental materials, resin infiltrant was applied for caries arrestment, which meet the principle of microinvasive therapies nowadays (Borges et al., 2017). With resin infiltration, demineralization of enamel is hampered, for the reason a diffusion barrier of acids produced by biofilm is created (Paris et al., 2020). A three-year follow-up clinical study revealed that resin infiltration reduced 65-90% of the risk of caries compared with varying self-applied non-invasive interventions alone (Paris et al., 2020). However, it was difficult for infiltration treatment when lesions extending into deeper enamel or even dentine: complete penetration is less reliable (Yim et al., 2014; Min et al., 2015). Furthermore, it has been reported that the application of resin infiltrant can only seal off around 30-60% of lesion depth (Yim et al., 2014; Min et al., 2015). In addition, enamel surfaces are constantly exposed to the oral microflora, many *in vitro* studies reported that there even was a hardness loss for the resin-infiltrated area after acid attack (Tawakoli et al., 2016). Common methacrylates in restorative materials, such as TEGDMA and BisGMA, do not possess strong antimicrobial activities (Flor-Ribeiro et al., 2019). To give the material antibacterial properties, researchers added antibacterial agents into resin infiltrant. Several studies modified the materials with AgNP (Kielbassa et al., 2020), quaternary ammonium methacrylate (Yu et al., 2020), ionic liquid (Cuppini et al., 2021), etc., which has potential results. But they had the same limitation, that is killing all the bacteria instead of showing an antibacterial effect only during microdysbiosis. They not only killed cariogenic bacteria, but also inhibited probiotics, the balance of oral eubiosis was affected once again (Liang et al., 2020; Li et al., 2021). Thus, a novel intelligent antibacterial resin infiltrant was needed.

We modified the resin infiltrant with DMAEM at the first time, which showed an antibacterial effect only during microdysbiosis. The total microorganisms were reduced by 1-4 logs. And the CFUs of *Mutans Streptococci* were reduced by 1-3 logs. Moreover, lactic acid production of the infiltrant + 5% DMAEM group was greatly reduced, which reduced by 92% compared to the infiltrant + 0% DMAEM group (control). Metabolic ability and biofilm accumulation were all reduced significantly with the increase of DMAEM concentration (*p* < 0.05). Therefore, the new intelligent resin infiltrant containing DMAEM are promising to inhibit biofilm growth. The pH-sensitive ability of DMAEM confers intelligent antibacterial properties to the novel resin infiltrant. DMAEM is a kind of intelligent materials, that could respond to the decrease of pH. In our previous work, DMAEM showed strong antibacterial effects when pH was 5. While when pH was 7.4, the MIC and MBC of DMAEM were more than 13.5, which showed lower antibacterial activity (Liang et al., 2020). Both *in vivo* and *in vitro* experiments indicated that in resin adhesives, DMAEM provided long-term antibacterial effect *via* its reversible pH-responsive activity (Liang et al., 2020). And DMAEM sealant demonstrated possibility to prevent microleakage in sealant application (Li et al., 2021). Also, it has been proved that DMAEM could maintain the diversity of oral microbiome, and was friendly to commensal microbiota due to its pH-sensitive activity (Liang et al., 2020). The reason why DMAEM showed antibacterial effect when pH was 5 is for the nitrogen atoms of DMAEM. In lower pH, the nitrogen atoms could be protonated to form QAMs, which showed strong antibacterial effect, while as pH increases, they are deprotonated and returned to DMAEM structure (Liang et al., 2020; Li et al., 2021). QAMs were proved strong antibacterial effect: the electrostatic interaction between the negatively-charged bacterial cell membrane and the positively-charged (N+) sites of a QAM resin causes the bacterium bursts; in addition, QAMs with long alkyl chains can physically pierce the bacterial cell wall, puncturing the cell membrane and releasing its cellular contents (Cheng et al., 2017). Therefore, DMAEM resin infiltrant could overcome the limitations of the present materials. Although DMAEM yielded covalently grafting to the resin infiltrant, which should have a long-term effect theoretically, future experiments are still needed to investigate the longevity of antibacterial effect.

Enamel surfaces are constantly exposed to the oral microflora (Arslan et al., 2015). Therefore, we used a saliva-derived biofilm to evaluate antibacterial effect and anti-demineralization effect of resin infiltrant. To simulate the conditions of caries, sucrose was added to the culture medium. Thus, cariogenic bacteria such as *S. mutans* and *Lactobacilli* metabolize carbohydrates to acids, causing demineralization of the tooth structure and the tooth-restoration margins beneath the biofilm (Cheng et al., 2012). Furthermore, to imitating an extreme clinical situation, and allowing demineralization to occur in a short time, the biofilms were cultured for 7 days continuously. The biofilm model successfully resulted in significant demineralization within 7 days, comparable to that with the use of a chemically-prepared acidic gel system for 21 days (Zhang et al., 2019). The biofilm model used in this study had main advantages over the traditional chemically-induced demineralization. It is more clinically relevant as it mimics a cariogenic situation, and it takes less time to construct a caries demineralization model.

Demineralization, which could reduce the enamel hardness, is an important process as well as outcome in the occurrence of WSLs. The microorganisms along the demineralized area could re-penetrate deeper, leading to further demineralization, and finally the cavities formed (Paris et al., 2013; Neres et al., 2017). Therefore, it is important to evaluate whether the resin infiltrant can inhibit demineralization. It has been proved that resin infiltrant could significantly increase both micro-hardness and demineralization resistance of enamel, which prevent or reduce the progression of caries (Brignardello-Petersen, 2020). Reported surface micro-hardness (352 HVN ≈ 3.45 GPa) for sound human enamel was consistent with sound bovine enamel hardness in our study; moreover, the hardness of natural carious enamel (0.29-3.29 GPa) was similar to hardness of artificial caries found in this study, either (Maupomé et al., 1998; Huang et al., 2010; Paris et al., 2013). After resin infiltration, the hardness of demineralized enamel recovered as sound enamel. It is doubtful whether the hardness could recover after resin infiltration. Some studies showed that the hardness of infiltrated enamel was less than sound enamel, while others showed increased hardness value (Maupomé et al., 1998; Huang et al., 2010; Paris et al., 2013; Dai et al., 2022). For instance, in Dai’s *in vitro* study, the enamel treated with ICON showed lower hardness (1.7 GPa) compared to sound enamel and experimental resin infiltrant containing TEGDMA and BisGMA (Dai et al., 2022). The reason may be that the ICON infiltrant possessed a polymer network mainly consisting of TEGDMA, which composition is likely to lead to lower hardness properties for the ICON infiltrant (Chen et al., 2019; Dai et al., 2022). Furthermore, BisGMA and TEGDMA based infiltrants may reduce polymerization shrinkage due to higher molecular weight (Paris et al., 2013). And except for the different materials, the operation could be another reason. For example, removing the excess infiltrant, polishing the surface, and the way to etch the surface could be different, that may influence the outcome (Zakizade et al., 2020). Then, after one-week biofilm attack, the hardness decreased again except for the infiltrant + 5% DMAEM group. In addition, the hardness value increases with the increase of DMAEM concentration. Our results indicated that under the cariogenic microbial environment, the resin infiltant may not be enough to resist demineralization. The possible reason for the continued decrease in the other groups is the continuous biofilm attack under an extremely severe cariogenic condition, for three reasons. First, unlike the oral environment, brushing teeth or chewing will partially eliminate the biofilm. Second, there wasn’t any buffer in the medium, and more sugar was added to mimic an environment prone to caries, and that was much more aggressive than what happened intraorally, where the saliva served as a buffer. Third, no source of minerals for remineralization was added in the medium, but there were minerals in the saliva intraorally. Considering resin infiltrant only seals the demineralized area, and the DMAEM resin infiltrant was a contact antibacterial material, future studies could combine release type of antimicrobial agent to further prevent the caries progression. Furthermore, although the experimental resin infiltrant was used in several studies, it did not outperform the commercial infiltrant (Paris et al., 2013), so more work needs to be done before it can be applied clinically.

Under these conditions, with the modification by DMAEM, the novel resin infiltrant showed comparable biocompability, mechanical properties, and strong antibacterial effect in an acidic environment. Moreover, these results demonstrated the anti-demineralization properties in the carious oral environment, which could prevent the progression of WSLs.

## 5 Conclusion

Development of novel dental materials that show an antibacterial effect only during microdysbiosis is an ideal way to inhibiting WSLs while maintaining a healthy oral eubiosis. In the present study, a novel pH-sensitive resin infiltrant containing DMAEM was synthesized for the first time. The new resin infiltrant presented good biocompability when the mass fraction of DMAEM was below 5% (*p* > 0.05). Biofilm metabolic activities, biofilm biomass, lactic acid production were substantially reduced, and biofilm CFU was reduced by up to 3 log. After acid attack by acid solution and then under by biofilms, the infiltrant + 5% DMAEM group produced an enamel hardness of 2.90 GPa, much higher than 0.85 GPa of the control infiltrant + 0% DMAEM group. Therefore, the novel intelligent resin infiltrant is highly promising for enamel infiltration to protect tooth structures and inhibit dental caries.

## Data availability statement

The original contributions presented in the study are included in the article/supplementary material. Further inquiries can be directed to the corresponding authors.

## Author contributions

XH and JL: Conceptualization, Methodology, Investigation, Writing - original draft. WZ, TM, and MW: Investigation. GH, GF, and TO: review & editing. HX and LC: Conceptualization, review & editing, project administration. XH, JL and LC: Funding acquisition. All authors contributed to the article and approved the submitted version.

## Funding

This study was supported by Chengdu Technological Innovation and R&D Project 2022-YF05-01415-SN (L.C); the Research Funding from West China School/Hospital of Stomatology Sichuan University, RCDWJS2021-19 (L.C.); China Postdoctoral Science Foundation Funded Project (519000-X92209); Research Funding from West China School/Hospital of Stomatology Sichuan University (No. RCDWJS2022-2).

## Conflict of interest

The authors declare that the research was conducted in the absence of any commercial or financial relationships that could be construed as a potential conflict of interest.

## Publisher’s note

All claims expressed in this article are solely those of the authors and do not necessarily represent those of their affiliated organizations, or those of the publisher, the editors and the reviewers. Any product that may be evaluated in this article, or claim that may be made by its manufacturer, is not guaranteed or endorsed by the publisher.

## References

[B1] Abdullah Z. John J. (2016). Minimally invasive treatment of white spot lesions–a systematic review. Oral. Health Prev. Dent. 14 (3), 197–205. doi: 10.3290/j.ohpd.a35745 26973988

[B2] Arslan S. Zorba Y. O. Atalay M. A. Ozcan S. Demirbuga S. Pala K.. (2015). Effect of resin infiltration on enamel surface properties and streptococcus mutans adhesion to artificial enamel lesions. Dent. Mater J. 34 (1), 25–30. doi: 10.4012/dmj.2014-078 25748455

[B3] Borges A. B. Caneppele T. M. Masterson D. Maia L. C. (2017). Is resin infiltration an effective esthetic treatment for enamel development defects and white spot lesions? a systematic review. J. Dent. 56, 11–18. doi: 10.1016/j.jdent.2016.10.010 27793705

[B4] Brignardello-Petersen R. (2020). Resin infiltration reduces the risk of experiencing proximal caries progression after 7 years. J. Am. Dent. Assoc. 151 (8), e67. doi: 10.1016/j.adaj.2020.02.005 32532520

[B5] Cheng L. Li J. Hao Y. Zhou X. (2008). Effect of compounds of galla chinensis and their combined effects with fluoride on remineralization of initial enamel lesion *in vitro* . J. Dent. 36 (5), 369–373. doi: 10.1016/j.jdent.2008.01.011 18308448

[B6] Cheng L. Weir M. D. Xu H. H. Antonucci J. M. Kraigsley A. M. Lin N. J.. (2012). Antibacterial amorphous calcium phosphate nanocomposites with a quaternary ammonium dimethacrylate and silver nanoparticles. Dent. Mater 28 (5), 561–572. doi: 10.1016/j.dental.2012.01.005 22305716 PMC3322309

[B7] Cheng L. Zhang K. Melo M. A. Weir M. D. Zhou X. Xu H. H. (2012). Anti-biofilm dentin primer with quaternary ammonium and silver nanoparticles. J. Dent. Res. 91 (6), 598–604. doi: 10.1177/0022034512444128 22492276 PMC3348066

[B8] Cheng L. Zhang K. Zhang N. Melo M. A. S. Weir M. D. Zhou X. D.. (2017). Developing a new generation of antimicrobial and bioactive dental resins. J. Dent. Res. 96 (8), 855–863. doi: 10.1177/0022034517709739 28530844 PMC5502962

[B9] Cheng L. Zhang K. Zhou C. C. Weir M. D. Zhou X. D. Xu H. H. (2016). One-year water-ageing of calcium phosphate composite containing nano-silver and quaternary ammonium to inhibit biofilms. Int. J. Oral. Sci. 8 (3), 172–181. doi: 10.1038/ijos.2016.13 27281037 PMC5113087

[B10] Chen M. Li J. Z. Zuo Q. L. Liu C. Jiang H. Du M. Q. (2019). Accelerated aging effects on color, microhardness and microstructure of ICON resin infiltration. Eur. Rev. Med. Pharmacol. Sci. 23 (18), 7722–7731. doi: 10.26355/eurrev_201909_18981 31599398

[B11] Cuppini M. Garcia I. M. de Souza V. S. Zatta K. C. Visioli F. Leitune V. C. B.. (2021). Ionic liquid-loaded microcapsules doped into dental resin infiltrants. Bioact. Mater 6 (9), 2667–2675. doi: 10.1016/j.bioactmat.2021.02.002 33665499 PMC7895677

[B12] Dai Z. Xie X. Zhang N. Li S. Yang K. Zhu M.. (2022). Novel nanostructured resin infiltrant containing calcium phosphate nanoparticles to prevent enamel white spot lesions. J. Mech. Behav. BioMed. Mater. 126, 104990. doi: 10.1016/j.jmbbm.2021.104990 34871957

[B13] Fenton O. S. Olafson K. N. Pillai P. S. Mitchell M. J. Langer R. (2018). Advances in biomaterials for drug delivery. Advanced materials (Deerfield Beach Fla.) 30 ,e1705328. doi: 10.1002/adma.201705328 PMC6261797 29736981

[B14] Flor-Ribeiro M. D. Graziano T. S. Aguiar F. H. B. Stipp R. N. Marchi G. M. (2019). Effect of iodonium salt and chitosan on the physical and antibacterial properties of experimental infiltrants. Braz. Oral. Res. 33, e075. doi: 10.1590/1807-3107bor-2019.vol33.0075 31432926

[B15] Gao Y. Liang K. Weir M. D. Gao J. Imazato S. Tay F. R.. (2020). Enamel remineralization *via* poly(amido amine) and adhesive resin containing calcium phosphate nanoparticles. J. Dent. 92, 103262. doi: 10.1016/j.jdent.2019.103262 31837358

[B16] Han Q. Li B. Zhou X. Ge Y. Wang S. Li M.. (2017). Anti-caries effects of dental adhesives containing quaternary ammonium methacrylates with different chain lengths. Materials (Basel) 10 (6), 643. doi: 10.3390/ma10060643 28773004 PMC5554024

[B17] Hashemian A. Shahabi S. Behroozibakhsh M. Najafi F. Abdulrazzaq Jerri Al-Bakhakh B. Hajizamani H. (2021). A modified TEGDMA-based resin infiltrant using polyurethane acrylate oligomer and remineralising nano-fillers with improved physical properties and remineralisation potential. J. Dent. 113, 103810. doi: 10.1016/j.jdent.2021.103810 34530057

[B18] He L. Deng D. Zhou X. Cheng L. ten Cate J. M. Li J.. (2015). Novel tea polyphenol-modified calcium phosphate nanoparticle and its remineralization potential. J. BioMed. Mater Res. B Appl. Biomater 103 (8), 1525–1531. doi: 10.1002/jbm.b.33333 25470574

[B19] Horst J. A. (2018). Silver fluoride as a treatment for dental caries. Adv. Dent. Res. 29 (1), 135–140. doi: 10.1177/0022034517743750 29355428 PMC6699125

[B20] Huang X. Ge Y. Yang B. Han Q. Zhou W. Liang J.. (2021). Novel dental implant modifications with two-staged double benefits for preventing infection and promoting osseointegration *in vivo* and *in vitro* . Bioact. Mater 6 (12), 4568–4579. doi: 10.1016/j.bioactmat.2021.04.041 34095616 PMC8141509

[B21] Huang T. T. He L. H. Darendeliler M. A. Swain M. V. (2010). Nano-indentation characterisation of natural carious white spot lesions. Caries Res. 44 (2), 101–107. doi: 10.1159/000286214 20173325

[B22] Huang X. Zhou W. Zhou X. D. Hu Y. Xiang P. Li B.. (2019). Effect of novel micro-arc oxidation implant material on preventing peri-implantitis. Coatings 9 (11), 691. doi: 10.3390/coatings9110691

[B23] Iizuka J. Mukai Y. Taniguchi M. Mikuni-Takagaki Y. Ten Cate J. M. Teranaka T. (2014). Chemical alteration by tooth bleaching of human salivary proteins that infiltrated subsurface enamel lesions–experimental study with bovine lesion model systems. Dent. Mater J. 33 (5), 663–668. doi: 10.4012/dmj.2014-046 25273046

[B24] Kielbassa A. M. Leimer M. R. Hartmann J. Harm S. Pasztorek M. Ulrich I. B. (2020). Ex vivo investigation on internal tunnel approach/internal resin infiltration and external nanosilver-modified resin infiltration of proximal caries exceeding into dentin. PloS One 15 (1), e0228249. doi: 10.1371/journal.pone.0228249 31990942 PMC6986723

[B25] Kielbassa A. M. Muller J. Gernhardt C. R. (2009). Closing the gap between oral hygiene and minimally invasive dentistry: A review on the resin infiltration technique of incipient (Proximal) enamel lesions. Quintessence Int. 40 (8), 663–681. 19639091

[B26] Leila B. Nemati S. Neda H. Khanehmasjedi M. (2017). The effect of MIpaste plus and reminpro on incipient caries using DIAGNOdent and SEM: An invitro study. J. Natl. Med. Assoc. 109 (3), 192–197. doi: 10.1016/j.jnma.2017.02.009 28987248

[B27] Liang J. Liu F. Zou J. Xu H. H. K. Han Q. Wang Z.. (2020). pH-responsive antibacterial resin adhesives for secondary caries inhibition. J. Dent. Res. 99 (12), 1368–1376. doi: 10.1177/0022034520936639 32600095

[B28] Liang K. Wang S. Tao S. Xiao S. Zhou H. Wang P.. (2019). Dental remineralization *via* poly(amido amine) and restorative materials containing calcium phosphate nanoparticles. Int. J. Oral. Sci. 11 (2), 15. doi: 10.1038/s41368-019-0048-z 31068570 PMC6506538

[B29] Li B. Ge Y. Wu Y. Chen J. Xu H. H. K. Yang M.. (2017). Anti-bacteria and microecosystem-regulating effects of dental implant coated with dimethylaminododecyl methacrylate. Molecules 22 (11), 2013. doi: 10.3390/molecules22112013 29156630 PMC6150392

[B30] Li H. Huang Y. Zhou X. Zhu C. Han Q. Wang H.. (2021). Intelligent pH-responsive dental sealants to prevent long-term microleakage. Dent. Mater 37, 1529–1541. doi: 10.1016/j.dental.2021.08.002 34412907

[B31] Li W. Qi M. Sun X. Chi M. Wan Y. Zheng X.. (2020). Novel dental adhesive containing silver exchanged EMT zeolites against cariogenic biofilms to combat dental caries. Microporous Mesoporous Materials 299, 110113. doi: 10.1016/j.micromeso.2020.110113

[B32] Li W. Zhou J. Xu Y. (2015). Study of the *in vitro* cytotoxicity testing of medical devices. BioMed. Rep. 3 (5), 617–620. doi: 10.3892/br.2015.481 26405534 PMC4535150

[B33] Maupomé G. Díez-de-Bonilla J. Torres-Villaseñor G. Andrade-Delgado L. C. Castaño V. M. (1998). *In vitro* quantitative assessment of enamel microhardness after exposure to eroding immersion in a cola drink. Caries Res. 32 (2), 148–153. doi: 10.1159/000016445 9580392

[B34] Min J. H. Inaba D. Kwon H. K. Chung J. H. Kim B. I. (2015). Evaluation of penetration effect of resin infiltrant using optical coherence tomography. J. Dent. 43 (6), 720–725. doi: 10.1016/j.jdent.2015.03.006 25862274

[B35] Neres E. Y. Moda M. D. Chiba E. K. Briso A. Pessan J. P. Fagundes T. C. (2017). Microhardness and roughness of infiltrated white spot lesions submitted to different challenges. Oper Dent. 42 (4), 428–435. doi: 10.2341/16-144-L 28402735

[B36] Paris S. Bitter K. Krois J. Meyer-Lueckel H. (2020). Seven-year-efficacy of proximal caries infiltration - randomized clinical trial. J. Dent. 93, 103277. doi: 10.1016/j.jdent.2020.103277 31931026

[B37] Paris S. Schwendicke F. Seddig S. Müller W. D. Dörfer C. Meyer-Lueckel H. (2013). Micro-hardness and mineral loss of enamel lesions after infiltration with various resins: influence of infiltrant composition and application frequency *in vitro* . J. Dent. 41 (6), 543–548. doi: 10.1016/j.jdent.2013.03.006 23571098

[B38] Paula A. B. Fernandes A. R. Coelho A. S. Marto C. M. Ferreira M. M. Caramelo F.. (2017). Therapies for white spot lesions-a systematic review. J. Evid Based Dent. Pract. 17 (1), 23–38. doi: 10.1016/j.jebdp.2016.10.003 28259311

[B39] Peres M. A. Macpherson L. M. D. Weyant R. J. Daly B. Venturelli R. Mathur M. R.. (2019). Oral diseases: a global public health challenge. Lancet 394 (10194), 249–260. doi: 10.1016/s0140-6736(19)31146-8 31327369

[B40] Pitts N. B. Zero D. T. Marsh P. D. Ekstrand K. Weintraub J. A. Ramos-Gomez F.. (2017). Dental caries. Nat. Rev. Dis. Primers 3, 17030. doi: 10.1038/nrdp.2017.30 28540937

[B41] Prodan D. Moldovan M. Chisnoiu A. M. Saroşi C. Cuc S. Filip M.. (2022). Development of new experimental dental enamel resin infiltrants-synthesis and characterization. Materials (Basel) 15 (3), 803. doi: 10.3390/ma15030803 35160748 PMC8836872

[B42] Rocha Gomes Torres C. Borges A. B. Torres L. M. Gomes I. S. de Oliveira R. S. (2011). Effect of caries infiltration technique and fluoride therapy on the colour masking of white spot lesions. J. Dent. 39 (3), 202–207. doi: 10.1016/j.jdent.2010.12.004 21172402

[B43] Rosier B. T. Marsh P. D. Mira A. (2018). Resilience of the oral microbiota in health: Mechanisms that prevent dysbiosis. J. Dent. Res. 97 (4), 371–380. doi: 10.1177/0022034517742139 29195050

[B44] Sardana D. Zhang J. Ekambaram M. Yang Y. McGrath C. P. Yiu C. K. Y. (2019). Effectiveness of professional fluorides against enamel white spot lesions during fixed orthodontic treatment: A systematic review and meta-analysis. J. Dent. 82, 1–10. doi: 10.1016/j.jdent.2018.12.006 30579859

[B45] Selwitz R. H. Ismail A. I. Pitts N. B. (2007). Dental caries. Lancet 369 (9555), 51–59. doi: 10.1016/s0140-6736(07)60031-2 17208642

[B46] Sonesson M. Brechter A. Abdulraheem S. Lindman R. Twetman S. (2020). Fluoride varnish for the prevention of white spot lesions during orthodontic treatment with fixed appliances: a randomized controlled trial. Eur. J. Orthod 42 (3), 326–330. doi: 10.1093/ejo/cjz045 31197364

[B47] Tawakoli P. N. Attin T. Mohn D. (2016). Oral biofilm and caries-infiltrant interactions on enamel. J. Dent. 48, 40–45. doi: 10.1016/j.jdent.2016.03.006 26972979

[B48] Wang L. Xie X. Qi M. Weir M. D. Reynolds M. A. Li C.. (2019). Effects of single species versus multispecies periodontal biofilms on the antibacterial efficacy of a novel bioactive class-V nanocomposite. Dent. Mater 35 (6), 847–861. doi: 10.1016/j.dental.2019.02.030 30878285

[B49] Wegehaupt F. J. Attin T. (2010). The role of fluoride and casein phosphopeptide/amorphous calcium phosphate in the prevention of erosive/abrasive wear in an *in vitro* model using hydrochloric acid. Caries Res. 44 (4), 358–363. doi: 10.1159/000316542 20668377

[B50] Wisniewski D. J. Ma T. Schneider A. (2021). Fatty acid synthase mediates high glucose-induced EGFR activation in oral dysplastic keratinocytes. J. Oral. Pathol. Med. Off. Publ. Int. Assoc. Oral. Pathologists Am. Acad. Oral. Pathol. 50 (9), 919–926. doi: 10.1111/jop.13235 PMC8530891 34402100

[B51] Yim H. K. Min J. H. Kwon H. K. Kim B. I. (2014). Modification of surface pretreatment of white spot lesions to improve the safety and efficacy of resin infiltration. Korean J. Orthod 44 (4), 195–202. doi: 10.4041/kjod.2014.44.4.195 25133134 PMC4130915

[B52] Yu J. Huang X. Zhou X. Han Q. Zhou W. Liang J.. (2020). Anti-caries effect of resin infiltrant modified by quaternary ammonium monomers. J. Dent. 97, 103355. doi: 10.1016/j.jdent.2020.103355 32380134

[B53] Zakizade M. Davoudi A. Akhavan A. Shirban F. (2020). Effect of resin infiltration technique on improving surface hardness of enamel lesions: A systematic review and meta-analysis. J. Evid Based Dent. Pract. 20 (2), 101405. doi: 10.1016/j.jebdp.2020.101405 32473796

[B54] Zhang K. Cheng L. Imazato S. Antonucci J. M. Lin N. J. Lin-Gibson S.. (2013). Effects of dual antibacterial agents MDPB and nano-silver in primer on microcosm biofilm, cytotoxicity and dentine bond properties. J. Dent. 41 (5), 464–474. doi: 10.1016/j.jdent.2013.02.001 23402889 PMC3654025

[B55] Zhang K. Cheng L. Wu E. J. Weir M. D. Bai Y. Xu H. H. (2013). Effect of water-ageing on dentine bond strength and anti-biofilm activity of bonding agent containing new monomer dimethylaminododecyl methacrylate. J. Dent. 41 (6), 504–513. doi: 10.1016/j.jdent.2013.03.011 23583528 PMC3751171

[B56] Zhang J. Lynch R. J. M. Watson T. F. Banerjee A. (2019). Chitosan-bioglass complexes promote subsurface remineralisation of incipient human carious enamel lesions. J. Dent. 84, 67–75. doi: 10.1016/j.jdent.2019.03.006 30951785

[B57] Zhang K. Melo M. A. Cheng L. Weir M. D. Bai Y. Xu H. H. (2012). Effect of quaternary ammonium and silver nanoparticle-containing adhesives on dentin bond strength and dental plaque microcosm biofilms. Dent. Mater 28 (8), 842–852. doi: 10.1016/j.dental.2012.04.027 22592165 PMC3393841

[B58] Zhou W. Peng X. Ma Y. Hu Y. Wu Y. Lan F.. (2019). Two-staged time-dependent materials for the prevention of implant-related infections. Acta Biomater 101, 128–140. doi: 10.1016/j.actbio.2019.10.023 31629895

[B59] Zhou W. Zhou X. Huang X. Zhu C. Weir M. D. Melo M. A. S.. (2020). Antibacterial and remineralizing nanocomposite inhibit root caries biofilms and protect root dentin hardness at the margins. J. Dent. 97, 103344. doi: 10.1016/j.jdent.2020.103344 32315666

[B60] Zou J. Meng M. Law C. S. Rao Y. Zhou X. (2018). Common dental diseases in children and malocclusion. Int. J. Oral. Sci. 10 (1), 7. doi: 10.1038/s41368-018-0012-3 29540669 PMC5944594

